# The Chisolm-Reuling Imbroglio

**Published:** 1880-10

**Authors:** 


### CORRESPONDENCE.

### THE CHISOLM-REULING EMBROGLIO.

Extra meetings of the State Medical Society were held on Friday 24th of September, to hear the report of the Committee on Ethics, who were instructed to investigate charges preferred against Dr. George Reuling by Dr. J. J. Chisolm, and what came of it. At the annual meeting of the Medico-Chirurgical Faculty, held in April last Dr. Reuling preferred charges against Dr. Chisolm for unprofessional conduct in inducing a patient to bring a law suit for mal-practice, with heavy damages; althought his statement had been disproved at the trial. The statement made by Dr. Reuling was based upon his report of a conversation held with Mr. Ruth, the husband of the lady who had lost her sight while under the care of Dr. Reuling. Mr. Ruth, both at the trial and before the Committee, denied most positively having had such a conversation with Dr. Reuling or of having mentioned the name of Dr. Chisolm in any way whatsoever to Dr. Reuling. It was proved by the report of the Ethical Committee that at the time of said conversation between Mr. Ruth and Dr. Reuling, none of the Ruth family had ever seen Dr. Chisolm, nor did he know that such a family as the Ruths existed.

The Committee, after a most thorough examination of many witnesses, reported back to the State Society that Dr. Reuling had no foundation whatever, for the accusation which he had made against Dr. Chisolm, and that on the contrary, ample testimony was shown in the examination of the legal counsel for the Ruths and others, that Dr. Chisolm used all means for preventing the trial.

When the report of the Committee of Ethics was made, animadverting upon the groundless and injurious charges made by Dr. Reuling, he sent in his letter of resignation, which the Society, by a large vote, laid on the table, on the ground that one who had made false accusations to injure a member, should not be allowed to withdraw. At a subsequent meeting of the State Society, Dr. Chisolm preferred charges against Dr. Reuling of devising a calumny, to be used at his second trial for influencing the jury, also for issuing and widely disseminating a pamphlet purporting to be a report of the second trial, in which the false accusations against Dr. Chisolm were reiterated. It is well known that these pamphlets have found their way into the houses of many private families. The State Society had instructed the Ethical Committee to investigate these charges. They had examined witnesses and were ready to report their findings at the extra meeting held September 24th, when Dr. Reuling presented a legal opinion from a distinguished jurist, and denied the jurisdiction of the Society to try him on these charges. He had sent in his letter of resignation before the charges had been made in writing and the Society had no right to refuse the reception of the resignation.

A very warm discussion ensued as to the right of a member to forestall the action of the Society and escape punishment by resigning when he feared that charges which he could not successfully meet would be preferred against him. The Society finally concluded that Dr. Reuling could not be legally held, as there was no by-law requiring the resignation to be accepted by the Society before it became valid. His resignation was therefore accepted. Notice was however at once given to so correct the constitution and By-Laws as to require a resignation to be accepted by the society before it took effect, so that in future members who by unprofessional conduct make themselves amenable to the society, can not creep out by the back door when wanted to answer for unethical and unprofessional conduct.

JUSTICE.

